# Attitudes of children and adolescents towards healthy lifestyle issues

**DOI:** 10.1093/eurpub/ckac131.018

**Published:** 2022-10-25

**Authors:** N Antia, D Tabidze, L Gabunia, I Zarnadze, Sh Khetsuraini, N Gamkrelidze, N Sulashvili

**Affiliations:** 1 Scientific Research Skills Center, Tbilisi State Medical University, Tbilisi, Georgia; 2 Department of Public Health, Tbilisi State Medical University, Tbilisi, Georgia

## Abstract

**Background:**

Health education for promoting healthy lifestyle from early ages is considered to be important. However, this issue has not been thoroughly examined in Georgia. The research, undertaken on adolescents, aimed to analyse adolescents’ lifestyle, awareness and attitudes towards healthy lifestyle.

**Methods:**

The study applied a mixed methods design and included both, qualitative and quantitative research methods. 145 students aged 17 to 23 years participated in the research. In order to evaluate the adolescent’s perceptions and attitudes, qualitative individual interviews with university students were conducted. For the quantitative method, a self-administered questionnaire assessing the level of adolescents’ awareness and attitudes towards healthy lifestyle was developed.

**Results:**

This section presents preliminary findings of the study. We found that more than 67% of adolescents were aware on the importance of healthy lifestyle. However, most of the participants did not transfer their knowledge into practice. The participants found it challenging to maintain healthy eating habits due to increased costs of healthy, organic food. The study also confirmed the need for integrated work of public, governmental, and non-governmental organizations to initiate and implement health promotion programs for children and adolescents.

**Conclusions:**

Overall, the study found that even though adolescents are aware about the importance of the healthy lifestyle, it is challenging for them to maintain it. In order to change adolescents’ attitudes towards unhealthy behaviour, it is necessary to carry out targeted interventions.

**Key messages:**

• Health education is a necessary component for healthy lifestyle.

• Healthy lifestyle requires careful study of the issue and making evidence-based conclusions.

